# CpG Type A Induction of an Early Protective Environment in Experimental Multiple Sclerosis

**DOI:** 10.1155/2017/1380615

**Published:** 2017-03-05

**Authors:** James Crooks, Marco Gargaro, Carmine Vacca, Claudia Volpi, Matteo Pirro, Giulia Scalisi, Antonella Turco, Rita Romani, Davide Matino, Abdolmohamad Rostami, Paolo Puccetti, Bruno Gran, Francesca Fallarino

**Affiliations:** ^1^Division of Clinical Neuroscience, University of Nottingham School of Medicine, Nottingham, UK; ^2^Department of Experimental Medicine, University of Perugia, Perugia, Italy; ^3^Department of Medicine, University of Perugia, Perugia, Italy; ^4^Department of Neurology, Thomas Jefferson University, Philadelphia, PA, USA; ^5^Department of Neurology, Nottingham University Hospitals NHS Trust, Nottingham, UK

## Abstract

Experimental autoimmune encephalomyelitis (EAE) is an inflammatory, demyelinating disease of the CNS that mimics human multiple sclerosis (MS), and it is thought to be driven by Th1 and Th17 myelin-reactive cells. Although adaptive immunity is clearly pivotal in the pathogenesis of EAE, with an essential role of CD4^+^ T cells, little is known of early, innate responses in this experimental setting. CpG-rich oligodeoxynucleotides (ODNs), typically found in microbial genomes, are potent activators of TLR9 in plasmacytoid dendritic cells (pDCs). In this study, we compared the effects of two types of CpG, namely, type A and type B, on EAE. We found that treatment with CpG type A ODN (CpG-A), known to induce high amounts of IFN-*α* in pDCs, significantly reduced disease severity in EAE, relative to controls (12.63 ± 1.86 versus 23.49 ± 1.46, resp.; *p* = 0.001). Treatment also delayed onset of neurological deficits and reduced spinal cord demyelination, while increasing the percentage of splenic regulatory (Foxp3^+^ CD4^+^) T cells. CpG-A likewise reduced the levels of IL-17 and IFN-*γ* in the CNS. Mechanistic insight into those events showed that CpG-A promoted a regulatory phenotype in pDCs. Moreover, adoptive transfer of pDCs isolated from CpG-A-treated mice inhibited CNS inflammation and induced disease remission in acute-phase EAE. Our data thus identify a link between TLR9 activation by specific ligands and the induction of tolerance via innate immunity mechanisms.

## 1. Introduction

One of the strategies used by mammalian cells to sense pathogens via innate and adaptive mechanisms implies selective recognition of unmethylated oligonucleotide sequences containing CpG motifs, particularly abundant in bacterial and viral, but not mammalian, genomic DNA [1]. Immunotherapeutic applications of CpG-ODN as TLR9 agonists include not only approaches to enhance immune responses, but also immunosuppressive strategies, as is the case for treating allergy [2] and autoimmunity [3]. Accordingly, activation of TLR9 by endogenous ligands has been reported to be protective in autoimmunity, and TLR9-deficient mice exhibit exacerbated symptoms of myelin oligodendrocyte glycoprotein- (MOG-) induced EAE [4], as well as an enhanced susceptibility to experimental type 1 diabetes [5]. A protective role for TLR9 in the pathogenesis of systemic lupus erythematosus has also been documented [6]. In EAE, an ambivalent role has been hypothesized for TLR9, with some reports suggesting a proinflammatory function [4] and others, instead, arguing for an immunoregulatory role in that TLR9 deficiency exacerbated EAE [7]. Any differential effects of distinct TLR9 ligands have never been investigated so far.

Distinct CpG-rich sequences are known to stimulate TLR9, with variations occurring in the number and location of the CpG dimers [8]. When targeted therapeutically, TLR9 is typically stimulated making use of synthetic oligodeoxynucleotides, containing one or more unmethylated CpG dinucleotides (CpG ODNs), within a phosphorothioate (PS) nuclease-resistant backbone for improved in vivo stability [9]. The recognition of CpG motifs is highly specific in that a single-base difference will affect the magnitude of the resulting immune response. This is underscored by the reduced ability of mouse-optimal CpG-ODN sequences to stimulate primate cells and vice versa, despite minor sequence differences [10]. Moreover, sequences encoding unmethylated CpG dinucleotides that vary in number, location, or precise sequence flanking the unmethylated CpG have been identified that can differentially activate the innate immune system [10]. Among those, three major classes of stimulatory CpG ODNs have been identified based on structural characteristics and activity on human peripheral blood mononuclear cells (PBMCs), in particular, B cells and plasmacytoid dendritic cells (pDCs), namely, Class A, Class B, and Class C CpG ODNs [11]. Type B ODNs (CpG-B) are the most extensively studied for their use as adjuvants in clinical trials. They possess PS backbones, encode multiple TCG (thymine-cytosine-guanine) motifs, and primarily stimulate B-cell and monocyte proliferation, as well as IgM, interleukin-10 (IL-10), and IL-6 secretion, with only marginal and transient secretion of IFN-*α* by pDCs [12]. In contrast, type A ODNs (CpG-A), with mixed phosphodiester and PS backbones, are the most potent inducers of IFN-*α* in pDCs [13]. The effects of the administration of type A or type B CpG ODNs in EAE have never been investigated.

The exact role of pDCs in the pathogenesis of MS is controversial, as they might have both pathogenic and protective functions. On the one hand, pDC-derived cytokines, including type I IFN and IL-6, will induce detrimental Th1 and Th17 cells, which are implicated in MS progression [14]. On the other hand, pDCs can exert a protective role in MS through the production of type I IFN [15]. Thus, although many questions remain, modulation of pDC functions as a means of controlling MS is currently the subject of intense investigation [16]. It has recently been demonstrated that when administered during the acute-phase of EAE, pDCs can lead to a dramatic remission [17]. Protection was indeed dependent on the transferred pDCs and correlated with reduced CNS inflammation and decreased abundance of encephalitogenic Th1 and Th17 cells [17]. In this study, we investigated any potential regulatory effects of two types of GpC ODNs (i.e., CpG-A and CpG-B) in EAE and their capacity to modulate innate immunity in this setting.

## 2. Materials and Methods

### 2.1. Mice

C57BL/6 mice were obtained from Charles River Breeding Laboratories. Female mice, 9-10-week-old, were used in all studies. All in vivo studies were in compliance with National (Italian Parliament DL 26/2014) and* Perugia University Animal Care and Use Committee* guidelines. All animal studies were approved by the Bioethics Committee of the University of Perugia. All animal experiments were approved by the University of Nottingham Animal Welfare and Ethical Review Body and performed in accordance with the UK Home Office License regulations, under Project License 40/3676.

### 2.2. EAE Induction

EAE was induced as described [18]. C57BL/6 mice received a subcutaneous (sc) immunization with 300 *μ*g of myelin oligodendrocyte glycoprotein fragment MEVGWYRSPFSRVVHLYRNGK (MOG35–55 peptide; Cambridge Research Biochemicals) in incomplete Freund's adjuvant (Difco Laboratories), containing 4 mg/ml* Mycobacterium tuberculosis* TB H37 Ra (Difco Laboratories). Two hundred nanograms of pertussis toxin (List Biological Laboratories, Inc) in 200 *μ*l PBS was injected intraperitoneally (i.p.) on the day of immunization and 2 days later. Mice were scored daily for clinical signs of EAE according to a standard scale: 0 = no symptoms; 1 = limp tail; 2 = partial paralysis of hind limbs; 3 = complete paralysis of hind limbs or partial hind and front limb paralysis; 4 = tetraparalysis; 5 = moribund/death [19].

### 2.3. In Vivo Treatment with CpG ODNs

Endotoxin-free CpG oligonucleotide, on a phosphorothioate backbone (CpG-B 5′ TCGTCGTTTTGTCGTTTTGTCGTT-3′) or on a phosphodiester backbone (CpG-A 5′-GGGGGATCGTCGGGGGG-3′), was purchased from Bio Fab Research. CpG (50 *μ*g) or vehicle was administered i.p., or intravenously (i.v.) in selected experiments, on days 1, 3, and 5 after immunization with MOG35–55 peptide for EAE induction. Vehicle-treated groups were used as controls.

### 2.4. Isolation of CNS Cells and Splenic Plasmacytoid DCs

Purification of brain-infiltrating leukocytes (BILs) from EAE animals was done as described [20]. Briefly, brains and spinal cord were recovered from anesthetized and cold PBS-perfused mice, and CNS cells were isolated by 70/30% Percoll gradient centrifugation (Sigma) by centrifugation at 300 ×g for 25 min. Infiltrating mononuclear cells were collected from the interface and washed with PBS.

pDC purification was conducted as previously described [21]. Briefly, spleens were harvested form mice with EAE, at the peak of the diseases (day 14 after immunization). pDCs were purified by magnetic-activated sorting in the presence of EDTA to disrupt DC–T-cell complexes using mpDCA-1 MicroBeads (Miltenyi Biotec, Bergisch Gladbach, Germany), after depleting B cells using CD19 microbeads (Miltenyi Biotec, Bergisch Gladbach, Germany). By this procedure, more than 95% of the mPDCA-1 cells were stained by 120G8 and expressed the B220 marker.

In the adoptive transfer of pDCs, mice were immunized and treated with CpG-A or vehicle as described above. These two groups of mice were used as donors of pDCs. A total of 5 × 10^5^ mPDCA1^+^ cells/mouse were transferred i.v. into recipients that had been immunized with the MOG35–55 peptide 4 days before adoptive transfer (day 0). After transfer, mice were scored daily for clinical signs of EAE.

### 2.5. Flow Cytometry

For flow cytometry analysis, cells were treated with anti-CD16/32 (clone 2.4G2; BD) for Fc receptor blockade and then stained with PE anti-CD4 (clone GK1.5; BD), Alexa Fluor 488 anti-Foxp3 (clone MF-14; Biolegend), or isotype control Alexa Fluor 488 rat IgG2b, as described [22]. FITC anti-CD11c (clone N418; e-Bioscience) and PE anti-CD11b (clone M1/70; BD) were also used. Analysis was done with an LSRFortessa (BD BioSciences) flow cytometer and analyzed by Flowjo data analysis software (Tree Star Inc).

### 2.6. Cytokine Measurements and Real-Time PCR Analysis

Splenocytes and brain-infiltrating lymphocytes (BILs) were cultured at a density of 2 × 10^6^/ml in IMDM medium containing 10% FCS in the presence of 25 *μ*g/ml MOG35–55 or 1 *μ*g/ml anti-CD3 (clone 2C11; BD) or PBS as a control. After 48 h of culture, cytokine concentrations were measured in supernatants by quantitative ELISA, according to manufacturer's recommendations. The range of detection for all cytokines was 10–5,000 pg/ml. Levels of IL-10, IL-17, IFN-*γ* (R&D Systems and BD), and IFN-*β* (Verikine) were measured in culture supernatant by ELISA using specific kits (R&D Systems and Abnova Corporation), according to manufacturer's recommendations. The detection limits (pg/ml) of the assays were 2 for IL-6, IL-10 and IL-17, and 15 for IFN-*β*.

Real-time PCR for mouse* Ido1* and* Gapdh* was carried out with specific primers as previously described [23]. Values were calculated as the ratio of* Ido1* to* Gapdh *expressions, as determined by the relative quantification method (ΔΔCT) (mean ± sem of triplicate determinations).

### 2.7. Histology and Immunohistochemistry

On day 40 after MOG immunization, mice were anesthetized and perfused transcardially with 4% paraformaldehyde in PBS. Spinal cords were removed, fixed for 24 h in ethyl alcohol (60%), acetic acid (10%), and chloroform (30%), and embedded in paraffin. Spinal cord sections were cut at 30 mm and stained with H&E to reveal CNS inflammatory infiltrates. For immunohistochemistry, 30 mm sections were first soaked in 3% hydrogen peroxide to block endogenous peroxidase activity and then stained with anti-CD4 (clone RM4-5; Abcam), followed by the appropriate biotinylated secondary antibody (Vector Laboratories) and streptavidin-HRP (Zymed). Controls consisted of isotype-matched antibodies. For myelin staining, slides were incubated with Luxol fast blue stain (e-bioscience) for 18 h at 56°C, according to the manufacturer's protocol. Analysis was performed using QWin image analysis software (Leica). Percentage of demyelination was calculated manually measuring all areas of grey matter and the area of demyelination using the following formula, (Area of demyelination/Total area of white matter) × 100.

### 2.8. Statistical Analysis

In the in vivo experiments, nonparametric Mann–Whitney *U* test was used for comparisons of clinical scores between different groups. Paired data were evaluated by Student's *t*-test. In selected experiments Kruskal-Wallis test was used. All in vitro determinations are means ± sem from at least three independent experiments, unless otherwise indicated. All *n* values were computed by power analysis to yield a power of at least 80% with an *α*-level of 0.05. GraphPad Prism version 6.0 (San Diego, CA) was used for all analyses.

## 3. Results

### 3.1. CpG-A Reduces EAE Severity to a Greater Extent Than CpG-B

**Figure 1:**
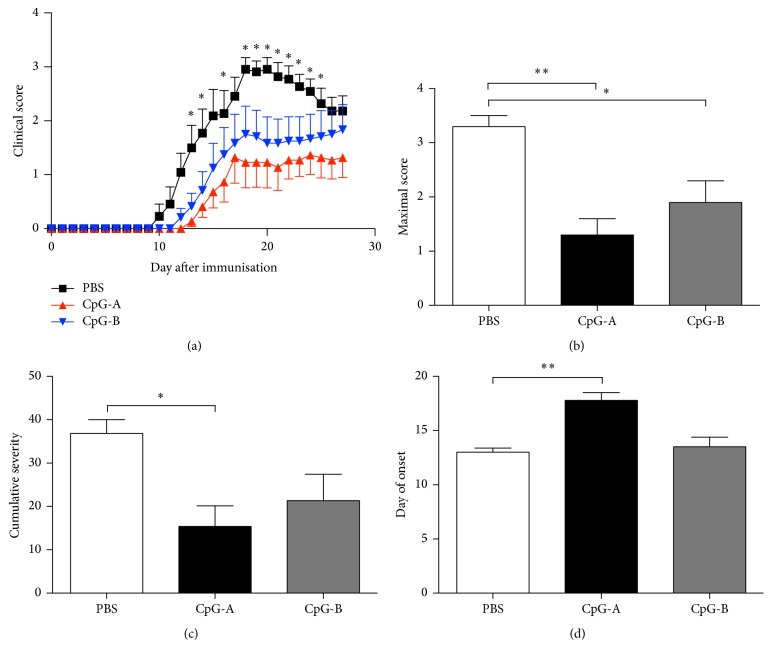
Effect of CpG-A and CpG-B on EAE. (a) Mice were i.p. treated with of CpG-A, CpG-B, or PBS on days 1, 3, and 5 p.i. Data are mean ± sem of daily scores, in one experiment representative of three independent experiments with *n* = 7 mice per group. ^*∗*^*p* < 0.05, (Mann–Whitney *U* test). (b) Intraperitoneal treatment with CpG-A reduced EAE maximal and cumulative scores and delayed the onset (d) in C57BL/6 mice when compared to PBS-treated mice. Depicted are the means ± sem from three separate experiments (*n* = 7 per group). ^*∗*^*p* < 0.05, by Kruskal-Wallis test followed by dunnett's test as post hoc. ^*∗∗*^*p* < 0.01.

TLR9 is known to be expressed on a number of cells, which play a pivotal role in innate and adaptive immune responses, including pDCs [24] and B cells [25]. To study the impact of two different CpG ODNs (i.e., CpG-A and CpG-B) on autoimmune CNS inflammation, we induced EAE in C57BL/6 mice by immunization with the MOG35–55 peptide in complete Freund's adjuvant (CFA). Immunized mice were then treated i.p. with three doses (50 *μ*g) of either CpG-A or CpG-B on days 1, 3, and 5, with control animals receiving PBS (Figure 1(a)). Only the CpG-A-treated group showed a significant reduction in disease severity, as compared to controls (Figures 1(a), 1(b), and 1(c)). Moreover, not only did CpG-A treatment reduce disease severity, but it also delayed clinical onset of EAE (Figure 1(d)). In contrast, despite a trend towards protection, the difference between CpG-B-treated and control animals was not significant (Figure 1(a)). Moreover, on testing two different routes of administration, we found that CpG-A significantly reduced disease severity only when given intraperitoneally but not when administered intravenously (see Supplementary Figure (1) in Supplementary Material available online at https://doi.org/10.1155/2017/1380615). CpG-B failed to reduce disease score by either route of administration (Supplemental Figure (1)). These results showed that the i.p. administration type A CpG, but not type B, modulates EAE severity.

### 3.2. CpG-A Reduces CNS Infiltration in EAE

**Figure 2:**
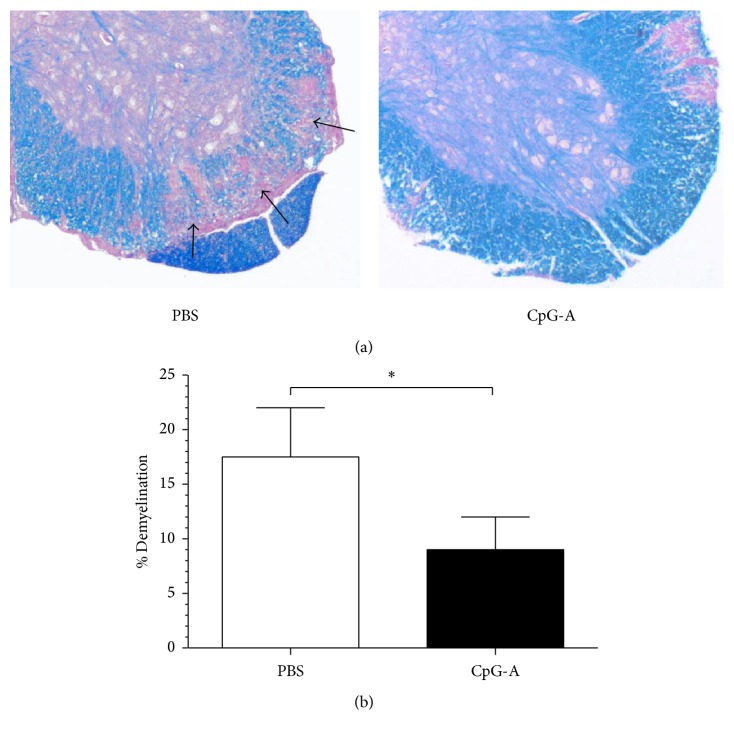
Decreased CNS demyelination in mice treated CpG-A during EAE. (a) Representative Luxol fast blue (LFB) staining of spinal cord sections from MOG-immunized mice treated with PBS or CpG-A to visualize demyelinization (arrows). Images are representative of one experiment out of three. (b) Quantification of spinal cord demyelination at day 20 after immunization in mice treated with PBS or CpG-A. Depicted are means ± sem in one representative of two. *n* = 5; ^*∗*^*p* < 0.05, two-tailed Mann–Whitney test.

**Figure 3:**
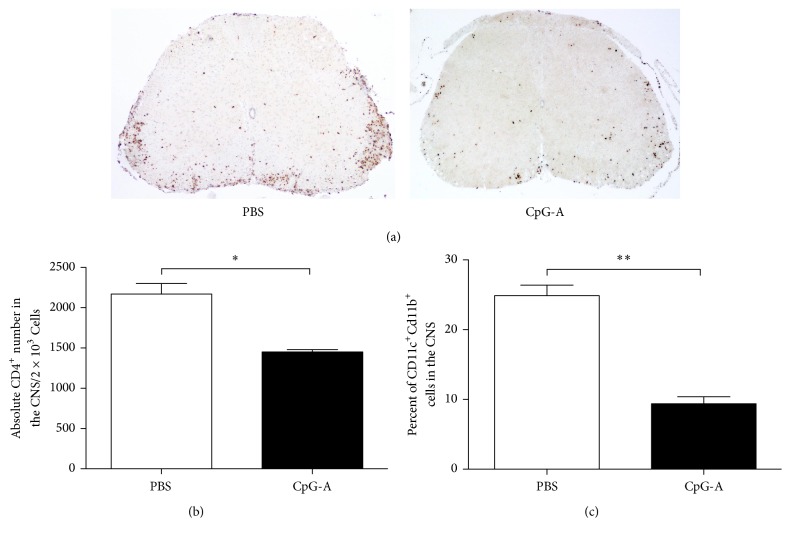
Decreased CNS lymphocyte infiltration in mice treated with CpG-A during EAE. (a) Representative images of spinal cross sections at peak of disease in specific groups (indicated). Stained CD4^+^ T cells are shown. Images are representative of one experiment out of three; *n* = 4 mice per group. Quantification of spinal cord immune-cell infiltration at day 20 after immunization in mice treated with PBS or CpG-A, CD4^+^ cells. (b) CD11b^+^/CD11c^+^. (c) Showed is the mean ± sem of two independent experiments; *n* = 7 per group. ^*∗*^*p* < 0.05, two-tailed Mann–Whitney test. ^*∗∗*^*p* < 0.01.

Based on the above results, we next focused solely on the effects of CpG-A treatment. Histopathological analysis revealed that the extent of demyelination and immune infiltrates in spinal cords at the peak of EAE disease were significantly reduced in the CpG-A-treated group relative to controls. Specifically, we found evidence for clear and significantly reduced demyelination in the spinal cords of CpG-A-treated mice as compared to PBS-treated controls (*p* = 0.034 CpG-A versus PBS) (Figures 2(a) and 2(b)). Similarly, the density of T lymphocytes (CD4^+^) in white matter was significantly reduced in CpG-A-treated mice at the peak of disease (*p* = 0.036; CpG-A versus PBS) (Figures 3(a) and 3(b)), providing further evidence for a reduction in CNS inflammation after CpG-A administration. In line with the clinical scores, the percentage of myeloid cells (mDCs; CD11c^+^ CD11b^+^) in the CNS was higher for control (24.9%) than CpG-A-treated animals (9.42%). Collectively, these results provided mechanistic insight into the cellular events underlying the beneficial effects of CpG administration in early EAE.

### 3.3. CpG-A Induces Regulatory Cytokines and Expansion of Treg Cells during EAE

**Figure 4:**
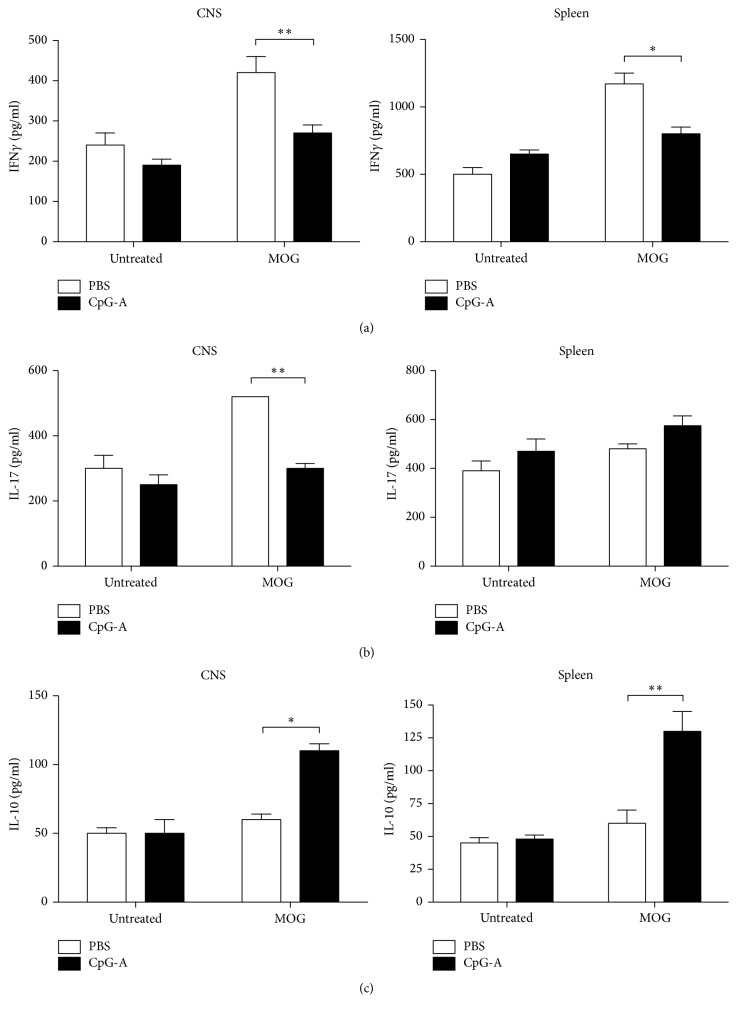
CpG-A treatment promotes regulatory cytokine responses. ELISA analysis of IFN-*γ* (a), IL-17 (b), and IL-10 (c) production from culture supernatants after MOG35–55 (25 *μ*g/ml) stimulation of CNS-infiltrating cells or splenocytes from PBS- or CpG-A-treated mice at the peak of disease. Results shown are means ± sem from three independent EAE experiments. ^*∗*^*p* < 0.05; ^*∗∗*^*p* < 0.01, two-tailed Mann–Whitney test.

**Figure 5:**
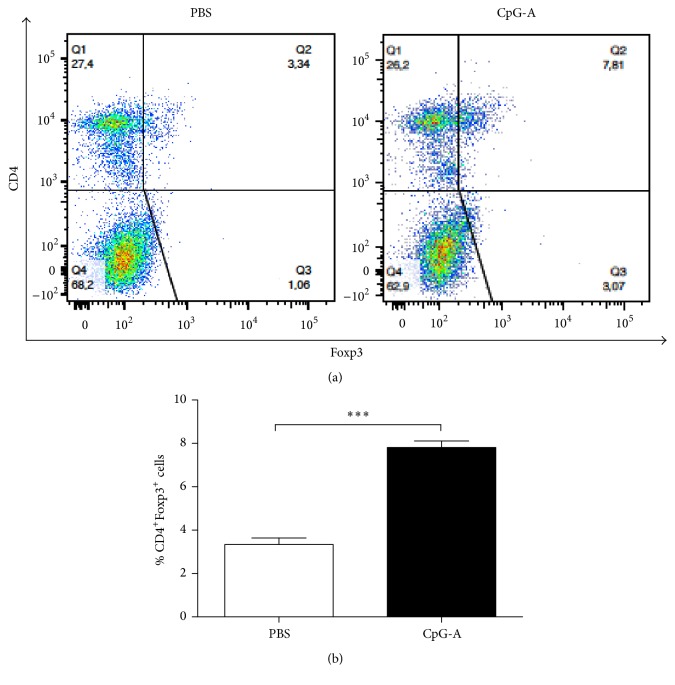
CpG-A treatment increases CD4+ Foxp3 Treg cell differentiation in EAE mice. (a) Flow cytometry analysis of CD4^+^ and Foxp3^+^ Treg cells in the spleen of specific groups (indicated) at the peak of disease. Numbers represent percentages of double positive cells. Data are representative of one of two independent experiments. (b) CD4^+^FoxP3^+^ T cell quantification in the spleens of mice treated with PBS or CpG-A. Mean ± sem; CpG-A versus PBS, ^*∗∗∗*^*p* < 0.001 and two-tailed Mann–Whitney test.

Th1 and Th17 responses are known to be associated with the proinflammatory phase of EAE, because of their production of specific cytokines, such as IFN-*γ* and IL-17 [26]. To assess any potential ability of CpG-A to suppress Th1 and Th17 responses, we measured cytokine production by brain lymphocytes and splenocytes, isolated from CpG-A- or PBS-treated EAE mice at the peak of diseases (i.e., on day 15 after immunization). Freshly harvested cells were restimulated with the MOG peptide or PBS, as a control, under in vitro conditions. We found a significant reduction in both IFN-*γ* and IL-17 productions by BILs from CpG-A-treated mice as compared to PBS receiving controls, when the cells were restimulated with MOG (Figures 4(a) and 4(b)). Splenocytes isolated from CpG-A treated mice produced significantly reduced the levels of IFN-*γ*, compared to splenocytes form PBS-injected controls (Figure 4(a)). Complementing the decreased production of proinflammatory cytokines, we also found that the in vivo treatment of EAE mice with CpG-A would result in an enhanced production of anti-inflammatory IL-10 by BILs and splenocytes when cells were restimulated in vitro as described above (Figure 4(c)). The reduction in immune-cell infiltrate, observed in CNS from CpG-A-treated mice, and the increase of IL-10, argued for the induction of a regulatory response in early EAE, as a result of CpG-A treatment. We measured CD4^+^ FoxP3^+^ (Treg) cell levels in the spleen of mice on CpG-A or PBS at the peak of disease. We found increased numbers of Treg cells after treatment with CpG-A relative to PBS-injected controls (Figure 5).

### 3.4. CpG-A Induces EAE-Suppressive Regulatory pDCs

**Figure 6:**
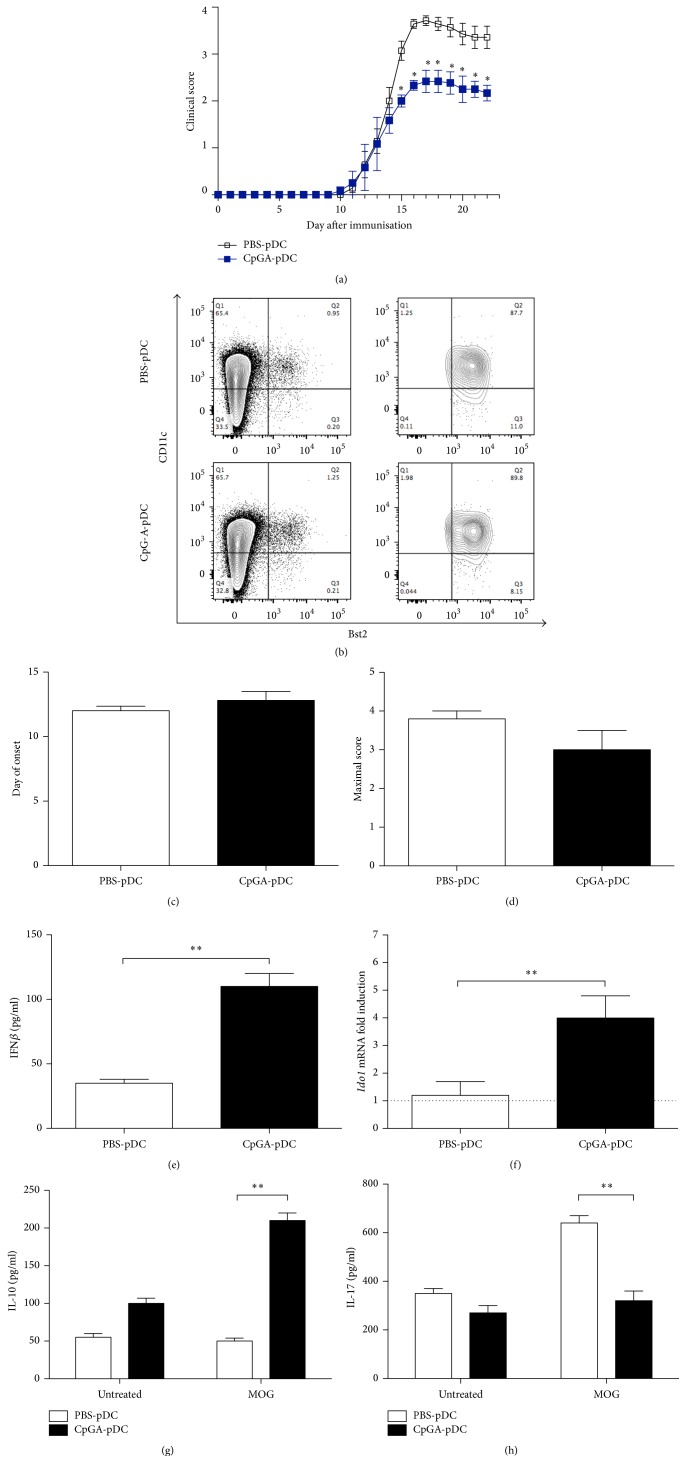
Clinical effect of adoptive transfer of pDCs from mice treated with CpG-A or PBS on EAE. (a) Mice received an i.v. injection of pDCs (5 × 10^5^ cells/mouse harvested from mice previously treated with CpG-A (Cp-GA-pDCs) or PBS (PBS-pDC)). Data represent average daily scores ± sem of two independent experiments; *n* = 7 per group, ^*∗*^*p* < 0.05; Mann–Whitney *U* test. Clinical parameters of disease scores following PBS-pDC or Cp-GA-pDCs treatment; (b) purity of pDC used for adoptive transfer. Numbers represent percentages of double positive cells. Data are representative of one of two independent experiments; (c) day of disease onset; (d) maximal score. Data represent mean ± sem from three independent experiments; *n* = 7. Mann–Whitney *U* test; (e) IFN-*β* production from pDCs harvested from PBS or CpG-A-treated animals and cultured for 24 h in vitro. Supernatants were tested for the presence of IFN-*β* via ELISA. Data are mean ± sem from two independent experiments; *n* = 7 per group. ^*∗∗*^*p* < 0.01. Mann–Whitney *U* test. (f)* Ido1* mRNA transcripts levels, expression of mRNA being presented relative to respective control, namely, freshly harvested pDCs (in which fold change = 1; dotted line). ^*∗∗*^*p* < 0.01. (g) Levels of IL-10 and IL-17 measured via ELISA following 72 h MOG (25 *μ*g/ml) culture of isolated splenocytes. Data represent mean ± sem from three independent experiments, with *n* = 7 mice per group; ^*∗∗*^*p* < 0.01 Mann–Whitney test.

pDCs are the main producers of type I interferons (IFN-I) in response to foreign nucleic acids, such as synthetic oligonucleotides, thereby indirectly influencing immunity [27]. To analyze whether CpG-A would promote regulatory functions in pDCs, we purified pDCs (B220^+^/pDCA1^+^) by means of magnetic sorting, from the spleens of EAE mice that had been treated with PBS (PBS-pDCs) or CpG-A (i.e., Cp-GA-pDCs). The sorted pDCs were transferred to C57BL/6 mice (5 × 10^5^/mouse), 4 days after MOG immunization. In recipients of PBS-pDCs, clinical scores continued to rise, whereas those of Cp-GA-pDCs-injected mice remained stable for a few days after transfer and leveling off, thereafter resulting in a significant inhibition of EAE on day 15 p.i. (Figure 6(a)). This correlated with a lower maximal score and a delayed disease onset (Figures 6(b), 6(c), and 6(d)). Also, in vitro, in accordance with the capacity of CpG-A to promote IFN-I production, we found a significant increase in the amount of IFN-*β* produced by Cp-GA-pDCs relative to PBS-pDCs, after 24 h stimulation with CpG-A (Figure 6(e)). This was associated with an increased transcriptional expression of the immunoregulatory enzyme indoleamine 2,3-dioxygenase 1 (IDO1) (Figure 6(f)). Reflecting the immunoregulatory function of the transferred pDCs, we found that splenocytes isolated from mice on Cp-GA-pDC immunotherapy produced higher IL-10 and lower IL-17 levels in response to MOG restimulation in vitro (Figures 6(g) and 6(h)). These results demonstrated that CpG-A acted on endogenous pDCs to confer protective regulatory functions on those cells and restrain early EAE.

## 4. Discussion

Different CpG types have been shown to affect immune responses in slightly different ways. Of interest, CpG type A has been reported to induce the production of large amounts of type I IFNs from pDCs, without affecting their maturation state [28]. CpG type B, on the other hand, has been shown to be a potent activator of B cells [29] and capable of inducing pDCs maturation and only marginally affecting pDC production of type I IFNs [13].

In this study, we assessed the effect of administration of these two different types of CpG, CpG-A, and CpG-B, on EAE. We found that CpG-A, but not CpG-B, significantly reduced severity of the disease and the extent of demyelination in EAE mice. Type-specific effects of CpG-B, such as the induction of pDC maturation and of IL-6 in several TLR9-expressing cell types, could account for the lack of beneficial activity by CpG-B in EAE immunotherapy [28, 30]. Because both protective and deleterious roles of TLR9 activation have been reported in EAE [4, 5], it is likewise possible that different TLR9 ligands initiate differential signaling events or recruit differential adaptor proteins, leading to ligand-specific immune responses. Notably, not only will A and B type CpGs induce different responses, but also, when applied simultaneously, they may exert opposing effects. Specifically, the B type blocks the CpG-A-induced secretion of IFN-*α*, and it may oppose the maturation of monocytes into DCs [29]. These observations suggest that A and B type CpG ODNs trigger mutually exclusive signaling pathways in TLR9-expressing cells, meaning that the signaling events triggered by one type of ODN precludes those cells from responding to the other [29].

The immunoregulatory action of CpG-A in EAE was further characterized in our study. We found that CpG-A administration during EAE clearly promoted a reduction in immune cells infiltrating the CNS, compared to vehicle-treated controls. Moreover, these cells were characterized by increased production of anti-inflammatory IL-10 and by decreased secretion of the proinflammatory cytokines IL-17 and IFN-*γ*, specifically in the CNS when analyzed at selected time points. Of note, the immunoregulatory function of CpG-A, as reported for a murine CpG-ODN [31], was dependent on route of administration, with the i.p. route significantly decreasing EAE scores compared to an i.v. administration. Although not addressed in this study, but as suggested by previous work [21, 23], it is also possible that CpG-A in EAE activates the noncanonical NF-*κ*B pathway, leading to* Ido1* induction in pDCs and triggering of the pDC-IDO1-Treg signaling pathway [32]. We found, indeed, that CpG-A-driven protection was associated by an increase in peripheral CD4^+^Foxp3^+^ Treg cells.

Crucial to the differential effects of A and B CpG is their ability to induce pDCs to secrete type I IFNs. Type B CpG induces low and short-lived levels of type I IFNs, whereas type A CpG will result in sustained production. Accordingly, we found that CpG-A administration promoted IFN-*β* production and* Ido1* induction in splenic pDCs. Notably, pDCs isolated from CpG-A-treated mice would confer EAE resistance on recipient mice, whereas control pDCs would not. These results are in accordance with previous results showing that CpG-A may promote immunomodulatory functions in specific TLR9-expressing cells, such as pDCs, due to the production of IFN-*β*. This mechanism may involve the activation of the IRF7 pathway [33].

Regulation of MyD88–IRF-7 signaling is critical for high-level IFN induction in response to TLR9 activation. The IFN-inducing TLR9 ligand, CpG-A, has been reported to be retained for long periods in the endosomal vesicles of pDCs, together with the MyD88-IRF-7 complex, all events representing a requirement for high-level production of type I interferons (IFN-*α*/*β*) [33]. Beneficial and deleterious effects have been assigned to IFN-*β* in CNS inflammation. Peripheral IFN-*β* administration is a first-line disease-modifying therapy for individuals with MS, and it is thought to act through its effects on DCs as well as B and T cells [34, 35]. Peripherally administered IFN-*β* does not cross the blood-brain barrier, but substantial amounts of IFN-*β* are produced by CNS-infiltrating by pDCs during EAE or by astrocytes and microglia [36]. Our data suggest that CpG-A-driven signaling events in pDCs result in pDC programming for therapeutic control of inflammation and immune-driven neurodegeneration. Thus, the study of specific CpG ODNs that regulate pDC activity may shed light on new mechanisms of pathogenesis and drive the development of more efficient therapeutic interventions in MS.

## 5. Conclusions

Our findings provide a novel mechanistic insight into an immunoregulatory role of different types of CpG-ODN in EAE. In particular, our data suggest that CpG-A-driven signaling events in pDCs result in pDC programming suitable for therapeutic control of inflammation and immune-driven neurodegeneration.

## Supplementary Material

In order to determine if the route of administration might have an impact on therapeutic activity of CpGA or CpGB, mice were treated i.p. or i.v. with of CpG-A, CpG-B, or PBS as control.
