# Supplementary figures and images for: CpG Type A Induction of an Early Protective Environment in Experimental Multiple Sclerosis
Source: Mediators Inflamm. 2017 Mar 5;2017:1380615. doi: 10.1155/2017/1380615 (PMC5357541; doi:10.1155/2017/1380615)

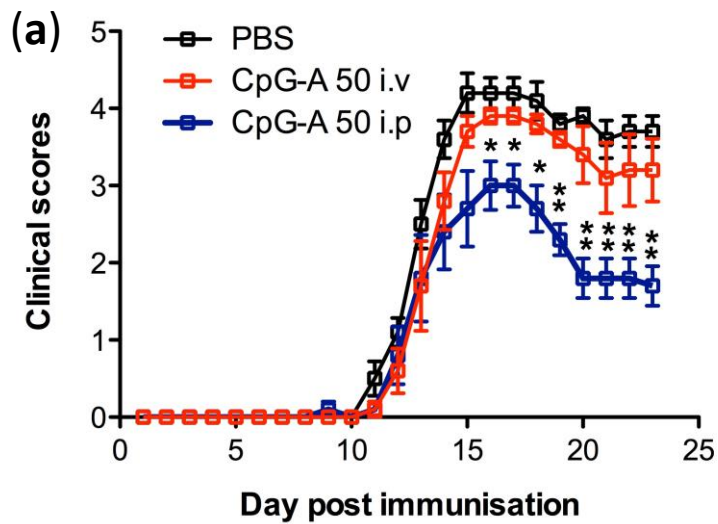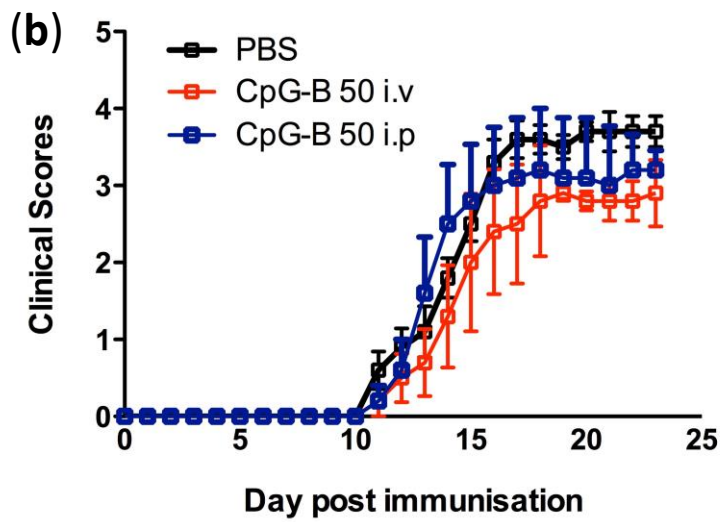

Supp Figure 1

Supplement: Supplementary file 1 — In order to determine if the route of administration might have an impact on therapeutic activity of CpGA or CpGB, mice were treated i.p. or i.v. with of CpG-A, CpG-B, or PBS as control. [file 1380615.f1.pdf]
